# Risk Indicators Improve the Prescription Quality of Drugs with Anticholinergic Properties in Nursing Homes

**DOI:** 10.3390/ijerph19010423

**Published:** 2021-12-31

**Authors:** Stéphane Sanchez, Jan Chrusciel, Biné Mariam Ndiongue, Caroline Blochet, Jean François Forget, Aude Letty, Paul Emile Hay, Jean Luc Novella

**Affiliations:** 1 Pole Territorial Santé Publique et Performance, Hôpitaux Champagne Sud, 10003 Troyes, France; Jan.chrusciel@ch-troyes.fr; 2 Fondation Korian Pour le Bien Vieillir, 75008 Paris, France; bine-mariam.ndiongue@korian.fr (B.M.N.); aude.letty@korian.com (A.L.); docteur.hay@gmail.com (P.E.H.); 3 EA 3797, Santé Publique, Vieillissement, Qualité de vie et Réadaptation des Sujets Fragiles, Université Reims Champagne Ardennes, 51100 Reims, France; jlnovella@chu-reims.fr; 4 Medissimo, 78300 Poissy, France; cblochet@medissimo.fr; 5 Science Department Vidal, 92130 Issy les Moulineaux, France; jean-francois.forget@vidal.fr; 6 Pole Autonomie Santé, Centre Hospitalier Universitaire de Reims, 92130 Issy les Moulineaux, France

**Keywords:** nursing homes, management indicators, anticholinergic, potentially inappropriate prescriptions

## Abstract

Aim: The objective of this study was to assess the impact of a collaborative therapeutic optimization program on the rate of potentially inappropriate prescription of drugs with anticholinergic properties in nursing homes. Methods: Quasi-experimental study in 37 nursing homes in France. The intervention included the use of quality indicators for prescriptions combined with educational sessions and dedicated materials for nursing home staff (unlimited access to study material for staff, including nurses, general practitioners, pharmacists). Indicators were calculated based on routine data collected from an electronic pill dispenser system. The primary outcome was the presence of at least one prescription containing ≥1 drug from a list of 12 drugs with anticholinergic properties. A difference-in-differences analysis was conducted at 18 months as well as propensity score weighting to minimize any potential indication bias. A generalized estimating equation model estimated the probability of being prescribed at least one target drug at any time during a 9-month period for each resident. Results: In total, 33 nursing homes (intervention group: *n* = 10; control group: *n* = 23) were included, totalling 8137 residents. There was a decrease in the use of drugs with anticholinergic properties over time in both groups, as well as a decline in the intervention group compared to the control group (Odds Ratio: 0.685, 95% CI: 0.533, 0.880; *p* < 0.01) that was attributable to the intervention. An estimated 49 anticholinergic properties drug prescriptions were avoided by the intervention. Conclusion: This study found that an intervention based on indicators derived from routine prescription data was effective in reducing use of drugs with anticholinergic properties prescriptions in nursing homes.

## 1. Introduction

Nursing home residents are at increased risk of iatrogenic complications due to potentially inappropriate medications (PIMs) and polypharmacy. PIMs correspond to drugs that should not be prescribed in a specific population (such as nursing home residents) because the risk of adverse events clearly outweighs the clinical benefit, and safer or more effective alternatives exist [1,2,3]. PIMs are associated with both the increased use of healthcare services and an impaired quality of life [4,5,6]. In France, residents are prescribed an average of eight different drugs per day and, in some studies, have been shown to be given more than 10 drugs every day [4,5,6,7]. Reducing the number of PIMs for nursing home residents is therefore a priority to improve the quality and safety of care in nursing homes.

In the past 20 years, it has been difficult to improve the quality of prescriptions due to the internal organization of nursing homes in France. Each resident is free to choose a general practitioner (GP), who may or may not have their practice in or near the location of a nursing home, resulting in many prescribers for each nursing home. Furthermore, although a medical director plays a key role in overseeing medical prescriptions for a facility, they cannot directly prescribe medications for residents. More than three-quarters of nursing homes in France also do not have a central pharmacy with a pharmacist responsible of prescriptions within the facility. Strong collaboration between nursing home directors, and prescribers and pharmacists across the local community may therefore be required to introduce innovative interventions aimed at improving the quality of medical prescriptions in nursing homes.

Drugs with anticholinergic properties are often prescribed in nursing homes and are associated with numerous adverse events and an increased risk of complications [8,9,10]. The side effects of anticholinergic medication in older people have been a topic of discussion since at least the 1980s. In reducing the number of PIMs, it is necessary to reduce the prescribing of this class of drugs. Various interventions have been tested to optimize the quality of medical prescriptions in this context, including physician training to increase topic awareness and improve collaboration amongst physicians, pharmacists and staff [11,12,13]. In a recent meta-analysis of 8 studies (4 randomized trials and 4 pre-post intervention studies) 2 of the randomized studies, and all four non-randomized studies reported a decrease in ACB after interventions to reduce anticholinergic burden in adults aged 65 and over in any clinical setting [14]. The studies included in this meta-analysis tested various approaches, including education, pharmacist-led medication review, home medication review, multidisciplinary team interventions and electronic prescribing. To date, no study has specifically evaluated such interventions in France. To this end, we designed an educational intervention comprising an educational component, as well as audit and feedback, all elements previously shown to be effective in reducing anticholinergic prescriptions [15,16].

The objective of this study was to assess the impact of a collaborative therapeutic optimization program on the rate of prescription of drugs with anticholinergic properties among nursing homes residents.

## 2. Methods

### 2.1. Study Design

A quasi-experimental, controlled, multicentre, observational study in nursing homes belonging to a private healthcare group in France was performed on the period between April 2016 and April 2019. All nursing homes belonging to the Korian group were eligible for participation, which was on a voluntary basis. The Korian group is a French company specialised in providing long-term care services to older people. They provide a wide variety of housing solutions and services, with over 1000 facilities across 7 European countries. Nursing homes were included if they were using the same electronic pill dispensing system (Mono28^®^, Medissimo, Poissy, France) and would continue to do so throughout the planned duration of the study, had a medical director, collaborated with a community pharmacy for drug supply, volunteered to participate in the study and had all prescribing physicians and members of the Geriatric Coordination Committee agree to participate. The intervention group comprised volunteer nursing homes where the intervention (i.e., the collaborative therapeutic optimization program) was implemented, and the control group included nursing homes where the intervention was not implemented. Participating nursing homes were recruited from March to May 2017.

### 2.2. Intervention-Exposure

The intervention (exposure) included multiple components. Firstly, educational sessions were organized, with individual meetings with the healthcare professionals providing care for the residents of each nursing home in the intervention group. These meetings were intended to convey educational messages about the dangers of anticholinergic drugs in older people, and to explain alternative strategies. This was complemented with the provision of interactive educational material, and a file listing alternative for the most common anticholinergic drugs. The educational material included posters alerting to the dangers of anticholinergic medications, a booklet describing good prescribing practices in older individuals; and a list of alternatives drugs with lower anticholinergic burden for use in various indications. Examples of posters displayed in the intervention nursing homes are given in Figures S1 and S2 (Supplementary Materials).

Finally, four quality indicators were established for feedback, namely:

- The overall proportion of patients receiving anticholinergics (numerator: number of patients receiving anticholinergics; denominator: total number of patients);
- The number of patients who received at least 1 drug with anticholinergic properties (numerator: number of patients receiving at one least 1 drug with anticholinergic properties; denominator: total number of patients);
- Among these patients, the total number of drugs with anticholinergic properties per patient (calculated as the total number of drugs with anticholinergic properties divided by the total number of patients);
- The average number of drug prescriptions per patient (calculated as the total number of all drugs prescribed divided by the total number of patients considered, i.e., all nursing home residents during the study period). The Quality Indicators were developed in collaboration with the medical directors of the intervention nursing homes. They were chosen because they can easily be retrieved from the information stored in the electronic pill dispensers, and also because they represent variables that are commonly reported as outcomes in other trials of interventions to reduce anticholinergic burden or prescription of anticholinergics in the literature.

The four quality indicators were automatically calculated from routine prescription data and sent monthly to the nursing home medical director who shared it with prescribing physicians during the study period of 18 months. Indicators from the nursing home as well as for the intervention group overall were presented to the medical directors at each participating facility. An example of a monthly quality indicator report is provided in Table S1 (Supplementary Material 1).

In the control group, nursing home medical directors were simply informed that the data from their electronic pill dispensing system was going to be used for research purposes. No indicators were provided to the medical directors and/or prescribers of control group nursing homes.

The listed drugs with anticholinergic properties targeted in this study were: alimemazine, amitriptyline, chlorpromazine, clomipramine, clozapine, cyamemazine, dexchlorpheniramine, hydroxyzine, levomepromazine, mequitazine, oxomemazine and periciazine. These 12 drugs were chosen for this study because they are all classed as risk level 3 (on a scale of 1 to 3, where 1 is low risk, 2 is intermediate risk and 3 is high risk) on 3 different scales used to assess anticholinergic drugs (namely the Anticholinergic Burden Scale (ABS) [17], Anticholinergic Risk Scale (ARS) [18], and Anticholinergic Drug Scale (ADS) [19]). Furthermore, all 12 drugs are widely used in nursing homes, and finally, for all of them (except clozapine), alternative therapies exist that carry a lower anticholinergic risk.

The primary outcome was any prescription of at least one of these drugs during the study period (i.e., if at any time, a patient was prescribed any one or more of the drugs on the list, then the primary outcome was considered to be met). A prescription corresponded to a single medication, prescribed by a physician to a patient for a maximum duration of 28 days. For example: “Aspirin 75 mg, 1 per day, for 28 days” is counted as one prescription. Some prescriptions had shorter durations (as low as one day)”.

### 2.3. Study Population

Nursing home residents were eligible for analysis if they were aged 75 years or older, taking at least one prescribed drug and who did not explicitly refuse participation after being adequately informed about the study parameters. For eligible residents under legal guardianship, their guardian/caregiver was asked for authorization.

### 2.4. Data Collection

The age, sex, and drug consumption data, including treatment start and end dates, dosage, presentation identifier code (PIC) and time of consumption of residents were extracted from the electronic pill dispensing system database in an anonymized format on a monthly basis. We recorded: sex, age (in categories), number of prescriptions with >1 anticholinergic drug, number of beds in the nursing home, number of nursing home residents, absenteeism, number of medical and non-medical staff (in full-time equivalents), bed occupancy, full-time temporary or permanent staff, mortality rate and turnover. For multivariable modelling, the data was aggregated into four 9-month periods. For each nursing home, mortality data, number of falls, and number of hospitalizations were also calculated using existing internal data systems but were not shared with the nursing homes. Mortality was calculated as the number of deaths observed during each period divided by the number of person-months at risk in the same period. No nominative data were recorded in this study. The difference-in-differences design makes it possible to adjust at the level of the nursing home, but also at the level of the individual residents. The variables are dichotomized for entry into the model.

### 2.5. Statistical Analysis

Statistics were presented using the mean and standard deviation (SD), or the median and interquartile range, or minimum and maximum values. For categorical data, the number and percentages were presented. Baseline characteristics of the intervention group and the control group were compared using the *t*-test for independent samples, the Mann-Whitney *U* test when the distribution of the variable was asymmetric, and the Chi-square test for categorical data. This comparison was performed to ensure that the assumptions for the difference-in-differences analysis were met. We conducted a difference-in-differences analysis [20] to estimate the effect of the intervention since the study was quasi-experimental and propensity score weighting was used to minimize indication bias [21]. The parallel trends assumption was verified visually, and the common shocks assumption was met by choosing nursing homes from the same organization who were all exposed to the same conditions during the study period, (with the exception of the intervention) [20]. The specific weighting used in this study is presented in a methodology article by Stuart [21] and was designed so that the DID propensity score method does not require the same patients to be present in the pre and post periods. Propensity score weighting was also performed to limit inclusion bias. Four propensity score groups were defined as Propensity Score Groups 1–4. A propensity score corresponds to the conditional probability of the outcome, given the covariates. It makes it possible to adjust for a number of covariates simultaneously, and thereby, balances the covariates between groups, which in turn reduces bias [22].

**Figure 1:**
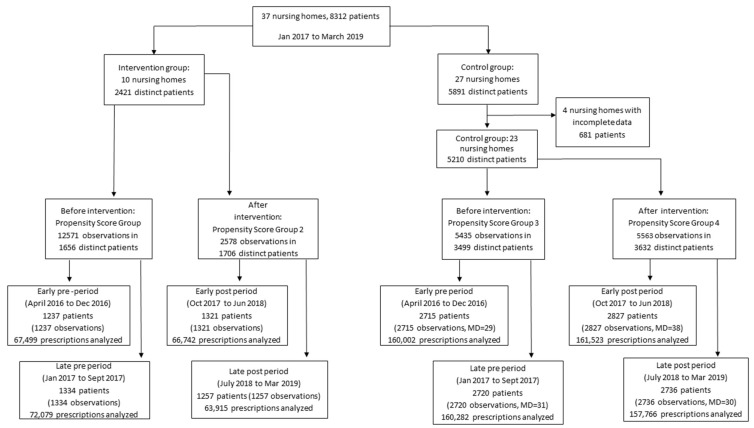
Flow chart of the study investigating the impact of the use of quality indicators on the quality of prescription of drugs with anticholinergic properties in nursing homes in France.

Nursing homes in the intervention group before the intervention were in Propensity Score Group 1 and those after the intervention were in Propensity Score Group 2. Nursing homes in the control group before the intervention were in Propensity Score Group 3 and those after the intervention were in Propensity Score Group 4. The flowchart of the nursing homes and number of residents is shown in Figure 1.

A multinomial logistic regression was used to model the probabilities of belonging to each of the four propensity score groups. The variables included in the regression were age, sex, number of beds and mortality rate of a nursing home. Observations were weighted to minimize indication bias [21]. Let *g* be the Propensity Score Group (1–4) of individual *i*. The weight for observation *i* was: wi = e1(Xi)/eg(Xi). where eg(Xi) represented the model-based probability of being in Group *g* for the individual *i* with covariates Xi. It followed that observations in Propensity Score Group 1 (assigned to treatment before treatment) were all assigned a weight of 1. The constitution of the propensity score groups is illustrated in Figure S3 (Supplementary Material S1).

A generalized estimating equation difference-in-differences model was also implemented to estimate the probability of having at least one prescription containing one or more anticholinergic drugs taken at any time during the study period per resident. The model included coefficients for period, treatment assignment group and the product term of their interaction. The model was adjusted for age, sex, number of beds in the nursing homes, medical full-time equivalents per bed, non-medical full-time equivalents per bed, absenteeism, bed occupancy, and the mortality rate of each nursing home during the period. The model was weighted to emphasize observations similar to Propensity Score Group 1. The hierarchical structure of the data was taken into account by using an exchangeable correlation structure for patients within nursing homes. *p*-values < 0.05 were considered statistically significant.

The point estimate of the number of events (prescriptions of anticholinergics) prevented by the intervention was approximated by calculating the Average Marginal Effect (AME) of the intervention, defined as the mean change induced by the interaction product term on the probability of the event for patients in the intervention group, using their observed characteristics as covariates.

Data management and statistical analyses were performed with R version 4.0.2 (www.r-project.org accessed on 27 December 2021).

### 2.6. Ethical Considerations

In accordance with French legislation, this was considered a non-interventional study with anonymous collected data. The electronic pill dispenser company holds authorization from the National Commission for the Protection of Privacy (Commission Nationale de l’Informatique et des Libertés, CNIL), under the number 1067312, for the use of their data for research purposes, in compliance with the European General Data Protection Regulation. All residents of participating nursing homes were informed of the study and were given the opportunity to refuse participation. The study was approved by the Ethics committee (Comité de Protection des Personnes Ouest 5 Rennes), under the number 20176A02862-51 in accordance with applicable French legislation relating to research involving human subjects.

## 3. Results

In total, there were 37 participating nursing homes, of which 33 were included. As shown in the study flowchart in Figure 1, 10 nursing homes were in the intervention group (2421 residents) and 23 in the control group (5210 residents). Overall, in the included nursing homes, data was missing in <1%. The characteristics of the nursing homes in the pre-intervention period are presented in Table 1. Covariates before and after propensity score weighting are presented in Table S2 and the balance plots in Figure S4 (see Supplementary Material 1).

Aggregated over 18 months before the intervention, there were a total of 320,284 prescriptions in the control group, and 139,578 in the intervention group, corresponding to 5.09 and 4.68 prescriptions per patient per month, respectively. Over an 18-month period after the intervention, on aggregate, there were 319,289 prescriptions in the control group, and 139,657 in the intervention group, corresponding to respectively 4.88 and 4.25 prescriptions per patient per month (Table S3, see Supplementary Material S1).

**Figure 2:**
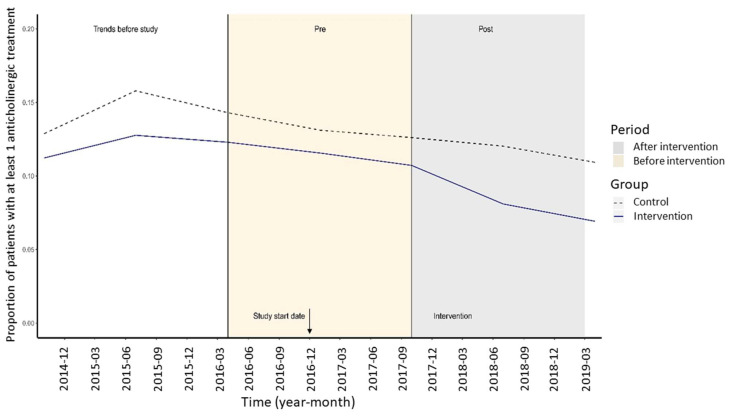
Trends before the study of potentially inappropriate prescriptions of drugs with anticholinergic properties (under rule 10) in nursing homes.

By univariate analysis, both groups displayed similar trends in anticholinergic prescriptions before the study period, as shown in Figure 2. The prescription of drugs with anticholinergic properties decreased over the study period in both groups (intervention and control), but the reduction was more marked in the intervention group. Results from the univariate and multivariable analyses are presented in Table 2. There was a significant difference between the two groups after the intervention (exponentiated interaction product term: 0.685, 95% CI: 0.533, 0.880; *p* < 0.01) indicating that the greater reduction observed in the intervention group was attributable to the intervention, independently of the effect of other variables.

An association between older age and the prescription of drugs with anticholinergic properties was also observed with residents aged 80 years and older being less likely to receive a prescription of drugs with anticholinergic properties compared to younger residents under the age of 80 years (Odds Ratio: 0.437, 95% CI: 0.348, 0.548; *p* < 0.001). Bed occupancy was also associated with decreased odds of having an anticholinergic prescription (Odds Ratio: 0.878, 95% CI: 0.785, 0.983; *p* = 0.02), although the smaller effect size and degree of significance suggest that this could be an incidental finding.

The AME was estimated to be −0.0189 on the response (probability) scale. Therefore, among 2578 observations of patients at risk during the last two 9-month periods, the number of anticholinergic drug prescriptions prevented by the intervention was estimated to be 49. Other anticholinergic prescription indicators are presented in Table S3 (Supplementary Material 1). Approximately 49 prescriptions of drugs with anticholinergic properties were estimated to have been prevented by the intervention.

## 4. Discussion

Our study found that the collaborative therapeutic optimization program implemented in the intervention group significantly decreased the prescription of drugs with anticholinergic properties for nursing home residents during the study period. Moreover, our study observed a reduction across a sample of nursing homes that are comparable to the national average of private for-profit nursing homes in France in terms of the number and mean age of residents which may suggest that a potential fall in anticholinergic drugs at a wider scale would be possible in France with generalization of our intervention14. In other words, the implementation of an intervention at a national level across all nursing homes in France could result in the prescribing of at least one less drug with anticholinergic properties to over 24,000 nursing home residents.

Our results are comparable to a similar intervention reported in an American study where a reduction in drugs with anticholinergic properties was subsequently observed (Odds Ratio: 0.95, 95% CI: 0.92, 0.98) [15]. In the American study, the training program toolkit included case-based lectures and clinical decision aids, but there was no reporting or feedback provided to participating nursing homes. There were also fewer residents and fewer prescribers overall than in our study.

Numerous studies have also shown the effect of targeted interventions comprising different health care professionals and methods of reducing the overall consumption of medications in nursing homes. In particular, interprofessional (nurse, pharmacist, general practitioner) interventions that involve medication review with a pharmacist [13,23]; specialised geriatric education for nursing home staff [24], auditing prescriptions based on geriatric risk assessment [25]; or retrospective drug utilization review activities have all been effective in improving the quality of prescriptions [26].

Our intervention differs from the ones cited previously by its focus on both the audit and feedback approaches. Providing monthly quality indicators to directors and prescribers allowed for an easy self-assessment of their own prescribing of anticholinergic drugs. Several reviews of related literature have demonstrated the efficacy of audit and feedback methods [27,28], while qualitative studies investigating the barriers and facilitators to their use have helped to clarify the specific features needing prioritisation [29,30]. By providing objective metrics summarizing both individual and collective performance, the use of feedback and quality indicators improved adherence to guidelines and standards and may have incited practitioners to improve their performance [31]. Social desirability bias, social pressure and competitiveness may also have contributed to enhancing quality in an environment favourable to self-assessment and self-improvement. A key advantage of our approach was that it required few resources for the collection and dissemination of indicators since they were automatically generated from routine prescription systems [32,33].

A decline in the prescribing of drugs with anticholinergic properties over time and independent of the other analysed factors was observed. This result matches with recent literature [34]. Similar reductions in the use of other drug classes have also been described in literature [35], and are encouraged by the national policy for medication use in nursing homes whose overall aim is to reduce drug consumption [33]. The choice of drugs was decided based on an analysis of the drug events and misuse most frequently observed in the nursing home group. This allowed the program to target drugs whose use was inappropriate with regards to the guidelines. The nursing homes in the intervention group had a lower rate of anticholinergic prescribing at the outset. This may be explained by the fact that they volunteered to participate in the intervention, suggesting that these may be nursing homes that are more mindful of the potential hazards of inappropriate prescriptions, and therefore, more attentive regarding the prescription of PIMs. However, the voluntary participation of the nursing homes in the study was in no way related to the prescribing practices of the GPs prescribing for the residents. Indeed, the GPs who prescribe for the residents are all independent, and are not employees of the nursing home. Consequently, this limits the potential for selection bias and indication bias, since both the control and intervention groups were exposed to a similar risk by a random sample of prescribing GPs. Therefore, we may assume that this imbalance in the rate of anticholinergic prescribing did not affect the results, especially since this factor was also accounted for by the propensity score.

Regarding limitations, our study was not randomized, which potentially induced selection and indication bias. However, a quasi-experimental study design was chosen to be pragmatic and test the hypothesis on a routine organizational approach. This bias was mitigated by using the propensity score weighting to balance any differences between groups. Another limitation could be that all participating nursing homes belonged to a single commercial operator and may not have been representative of all nursing homes settings in France. Nonetheless, this study allowed us to have comparable nursing homes and residents presented in the intervention.

In addition, the implementation method of the intervention within each nursing home was not standardised and was defined by the medical directors, which created heterogeneity between nursing homes in this regard. A significant reduction in drug with anticholinergic properties, however, was observed, indicating that the objective was reached despite the potentially heterogeneous implementation methods. Furthermore, the clinical impact of the reduction in drugs with anticholinergic properties prescription was not evaluated, as it was not feasible based on the data available for the study. These outcomes are indeed important for the residents, as they represent the ultimate benefit to be derived by the residents from the reduction of anticholinergic drugs. However, we did not have data about falls and hospitalizations suitable for analysis. Furthermore, the present study was designed to demonstrate that the intervention was associated with a significant reduction in prescription of drugs with anticholinergic properties, and could not allow any conclusions regarding the causal link between the reduction in prescriptions observed here, and clinical outcomes. Future studies are warranted to assess whether the reduction in prescriptions translates into clinical benefits. Finally, our study was limited to 12 drugs with anticholinergic properties among those most commonly prescribed in nursing homes. This choice could minimize the effect of the intervention, which may in fact have been greater, considering the large number of other drugs with anticholinergic properties.

## 5. Conclusions and Implications

Our study demonstrated the feasibility of using routine data from electronic pill dispensers to measure medication consumption and build quality indicators. Using these indicators to provide feedback to prescribers in combination with educational sessions and materials using a collaborative approach was effective in reducing the prescription of drugs with anticholinergic properties in nursing homes in the intervention group. These findings are relevant to improving drug prescription practices for older adults and to reducing their risk of iatrogenic complications.

## Figures and Tables

**Table 1:** Characteristics of the nursing homes, residents, and staff at baseline (pre-intervention period).

	Intervention Group *	Control Group *	*p*-Value
Number of nursing homes	10	23	-
Number of nursing home residents	1656	3499	-
Number of patients with ≥1 anticholinergic drug during pre-intervention period: mean (SD)	210 (12.7)	500 (14.3)	0.13
Male sex of residents; *n* (%)	593 (35.8)	1144 (32.7)	0.03
Age ≥ 80 years of residents; *n* (%)	1433 (86.5)	3107 (88.8)	0.02
Number of beds; median [Q1, Q3]	86.50 [84.00, 101.00]	92.00 [80.00, 101.00]	0.95
Absenteeism; mean (SD)	0.10 (0.03)	0.10 (0.04)	0.06
Full-time or equivalent medical staff; mean, (SD)	27.70 (5.75)	25.55 (6.07)	<0.001
Full-time or equivalent non-medical staff; median [Q1, median Q3]	16.00 [12.00, 18.75]	16.40 [13.49, 20.50]	0.003
Bed occupancy; median [Q1, Q3]	0.97 [0.96, 0.99]	0.96 [0.93, 0.99]	0.01
Full-time or equivalent temporary workers; mean (SD)	12.49 (3.55)	12.48 (4.86)	0.62
Full-time or equivalent permanent staff; mean (SD)	50.71 (8.95)	50.07 (9.74)	0.48
Mortality rate; median [Q1, Q3]	2.00 [1.00, 3.00]	2.00 [1.00, 3.00]	0.03
Turnover; median [Q1, Q3]	0.15 [0.10, 0.20]	0.15 [0.10, 0.21]	0.84

* Summary of a month-level nursing home data over study period unless otherwise specified. Excludes four nursing homes that stopped participating in the study resulting in incomplete data.

**Table 2:** Multivariable model by logistic regression modelling the probability of any prescription, including at least one drug with anticholinergic properties.

Variable	Unadjusted Analysis ^†^				Fully Adjusted PS-Weighted Analysis ^‡^			
	Odds Ratio	95% CI		*p*-Value (Wald Test)	Odds Ratio	95% CI		*p*-Value (Wald Test)
Period *				0.04 ^§^				0.41 ^§^
2016-04 to 2016-12 (baseline)	1	(Reference)			1	(Reference)		
2017-01 to 2017-09 (second pre-period)	0.947	0.828	1.084		0.927	0.810	1.062	
2017-10 to 2018-06 (early intervention period)	0.910	0.783	1.057		0.894	0.762	1.049	
2018-07 to 2019-03 (late intervention period)	0.805	0.690	0.939		0.848	0.698	1.030	
Intervention group (Reference: control group)	0.850	0.734	0.985	0.03	0.826	0.635	1.075	0.16
Effect of intervention in the intervention group: interaction product term	0.737	0.589	0.921	<0.01	0.685	0.533	0.880	<0.01
Male sex (Reference: female)	1.069	0.964	1.186	0.21	1.024	0.886	1.183	0.75
Age ≥ 80 years (Reference: <80 years)	0.442	0.390	0.501	<0.001	0.437	0.348	0.548	<0.001
Mortality rate in the nursing home during period ||	1.000	0.953	1.051	0.97	0.943	0.882	1.009	0.09
Number of beds ||	1.020	0.968	1.068	0.50	1.037	0.893	1.204	0.64
Bed occupancy ||	1.010	0.960	1.059	0.74	0.878	0.785	0.983	0.02
Medical full-time equivalents per bed ||	0.954	0.908	1.002	0.06	0.997	0.863	1.151	0.96
Non-medical full-time equivalents per bed ||	1.040	0.992	1.092	0.10	0.987	0.850	1.147	0.87
Absenteeism ||	0.912	0.868	0.958	<0.01	0.954	0.883	1.032	0.24

PS, Propensity Score; CI, Confidence Interval; DID, difference-in-differences; * both intervention and control groups considered together. ^†^ For the period, group, and DID variables, unadjusted analysis included the aforementioned main effects and the interaction term but excluded other variables. For sex, age, mortality rate, and number of beds, each unadjusted analysis included single independent variables. ^‡^ Generalized Estimating Equations logistic regression model with exchangeable working correlation matrix. ^§^ Overall *p*-value for all modalities of the time main effect. || Standardised (centered and scaled to unit standard deviation) quantitative variable.

## Data Availability

Data available on request due to restrictions eg privacy or ethical. All data material can be accessed upon request to the first author Dr Stéphane Sanchez at the following email address stephane.sanchez@hcs-sante.fr.

## Supplementary Materials

The following supporting information can be downloaded at: https://www.mdpi.com/article/10.3390/ijerph19010423/s1, Figure S1: Example of communication 1 displayed in the common areas; Figure S2: Example of communication 2 displayed in the common areas; Figure S3: Constitution of the propensity score groups; Figure S4: Balance plot before and after the intervention periods; Table S1: Example of quality indicators sent to the nursing homes in the intervention group every month; Table S2: Balance of the covariates before and after the propensity-score weighting; Table S3: Supplementary anticholinergic prescription indicators (aggregated over 18-month periods); Table S4: STROBE Statement—checklist of items that should be included in reports of observational studies.
