# Supplementary material for: Risk Indicators Improve the Prescription Quality of Drugs with Anticholinergic Properties in Nursing Homes
Source: Int J Environ Res Public Health. 2021 Dec 31;19(1):423. doi: 10.3390/ijerph19010423 (PMC8744764; doi:10.3390/ijerph19010423)
Supplement: Supplementary file 1 [file ijerph-19-00423-s001.zip › ijerph-1462980-supplementary.pdf]

Figure S1: Example of communication 1 displayed in the common areas

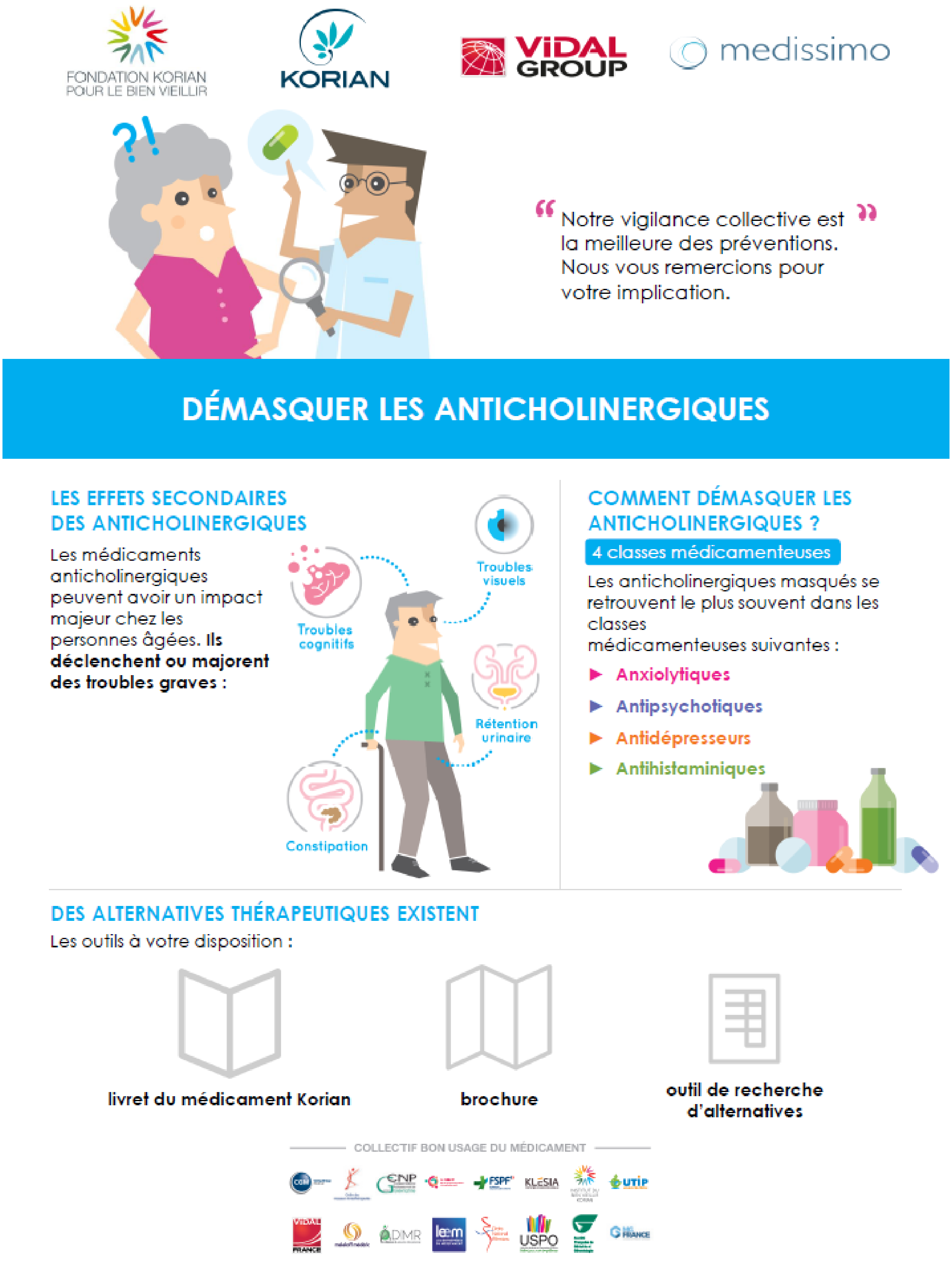

Figure S2: Example of communication 2 displayed in the common areas

DÉMASQUER LES ANTICHOLINERGIQUES

Comment les démasquer ?

4 classes médicamenteuses

Les anticholinergiques masqués se retrouvent le plus souvent dans les classes médicamenteuses suivantes :

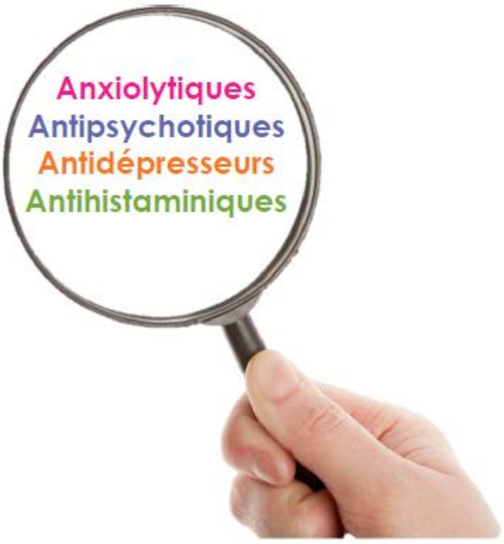

Les effets secondaires des Anticholinergiques

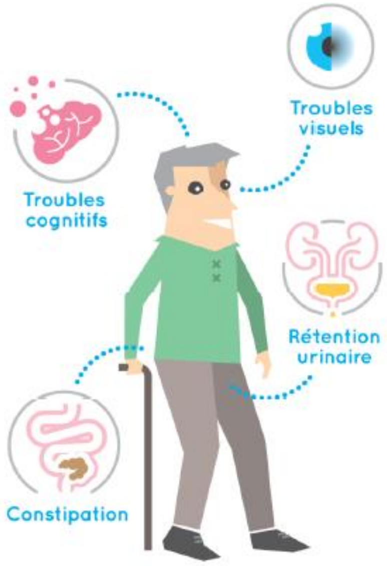

« Notre vigilance collective est la meilleure des préventions. Nous vous remercions pour votre implication. »

COLLECTIF BON USAGE DU MÉDICAMENT

DES ALTERNATIVES THÉRAPEUTIQUES EXISTENT
RAPPROCHEZ-VOUS DU MÉDECIN COORDONNATEUR

- livret du médicament Korian
- brochure
- outil de recherche d'alternatives

Table S1: Example of quality indicators sent to the nursing homes in the intervention group every month.

DemAsCH Study

FONDATION KORIAN POUR LE BIEN VIEILLIR

Key Indicators - February 2019

Indicator	Result for your centre
Proportion of nursing home residents receiving anticholinergics (%)	16
Number of residents who received at least one anticholinergic drug	16
Number of residents on site	101
Average number of anticholinergics per resident	2.00
Average number of prescriptions per resident	2.52

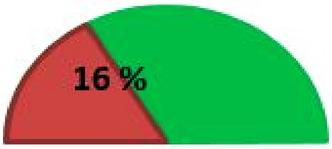

Use by molecule: In colour, the most prescribed anticholinergic drugs

HYDROXYZINE	ATARAX®	0	0.00%
CHLORPROMAZINE	LARGACTIL®	0	0.00%
LEVOMEPROMAZINE	NOZINAN®	1	6.25%

CYAMEMAZINE	TERCIAN®	1	6.25%
PERICIAZINE	NEULEPTIL®	0	0.00%
CLOZAPINE	LEPONEX®	0	0.00%
CLOMIPRAMINE	ANAFRANIL®	0	0.00%
AMITRIPTYLINE	ELAVYL®/LAROXYL®	2	12.50 %
ALIMEMAZINE	THERALENE®	0	0.00%
OXOMEMAZINE	TOPLEXIL®	0	0.00%
MEQUITAZINE	PRIMALAN®	0	0.00%
DEXCHLORPHENIRAMINE	POLARAMINE®	0	0.00%

Figure S3 : Constitution of the propensity score groups

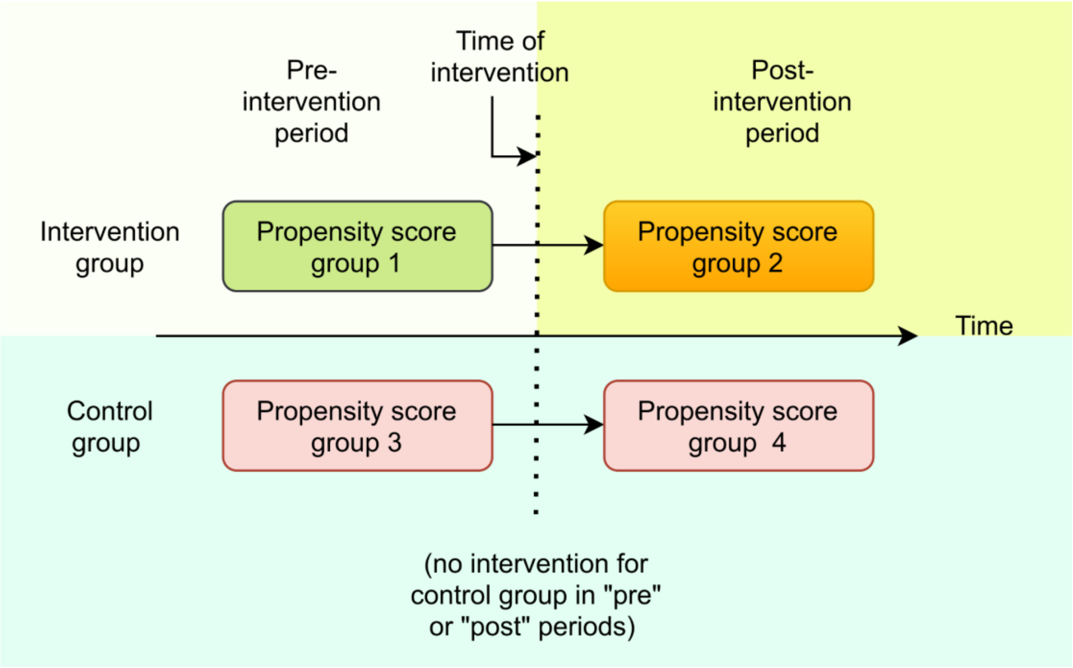

Table S2: Balance of the covariates before and after the propensity-score weighting

	Unweighted Standardized Difference in Means			Weighted Standardized Difference in Means		
	**Group 1* vs Group 2†**	**Group 1 vs Group 3‡**	**Group 1 vs Group 4§**	**Group 1 vs Group 2**	**Group 1 vs Group 3**	**Group 1 vs Group 4**
**Age (≥80)**	0.010	0.059	0.030	0.014	0.001	0.042
**Sex**	0.040	0.068	0.063	0.027	0.005	0.013
**Mortality**	0.014	0.148	0.322	0.002	0.154	0.122
**Number of beds**	0.005	0.218	0.232	0.124	0.048	0.086
**Medical full-time equivalents per bed**	0.237	0.378	0.408	0.046	0.243	0.228
**Non-medical full-time equivalents per bed**	0.037	0.522	0.556	0.086	0.255	0.224
**Bed occupancy**	0.485	0.364	0.681	0.360	0.150	0.054
**Absenteeism**	0.475	0.083	0.095	0.145	0.045	0.004

*Group 1: Intervention group before intervention; †Group 2: Intervention group after intervention; ‡Group 3: Control group before intervention; §Group 4: Control group after intervention

Figure S4: Balance plot before and after the intervention periods.

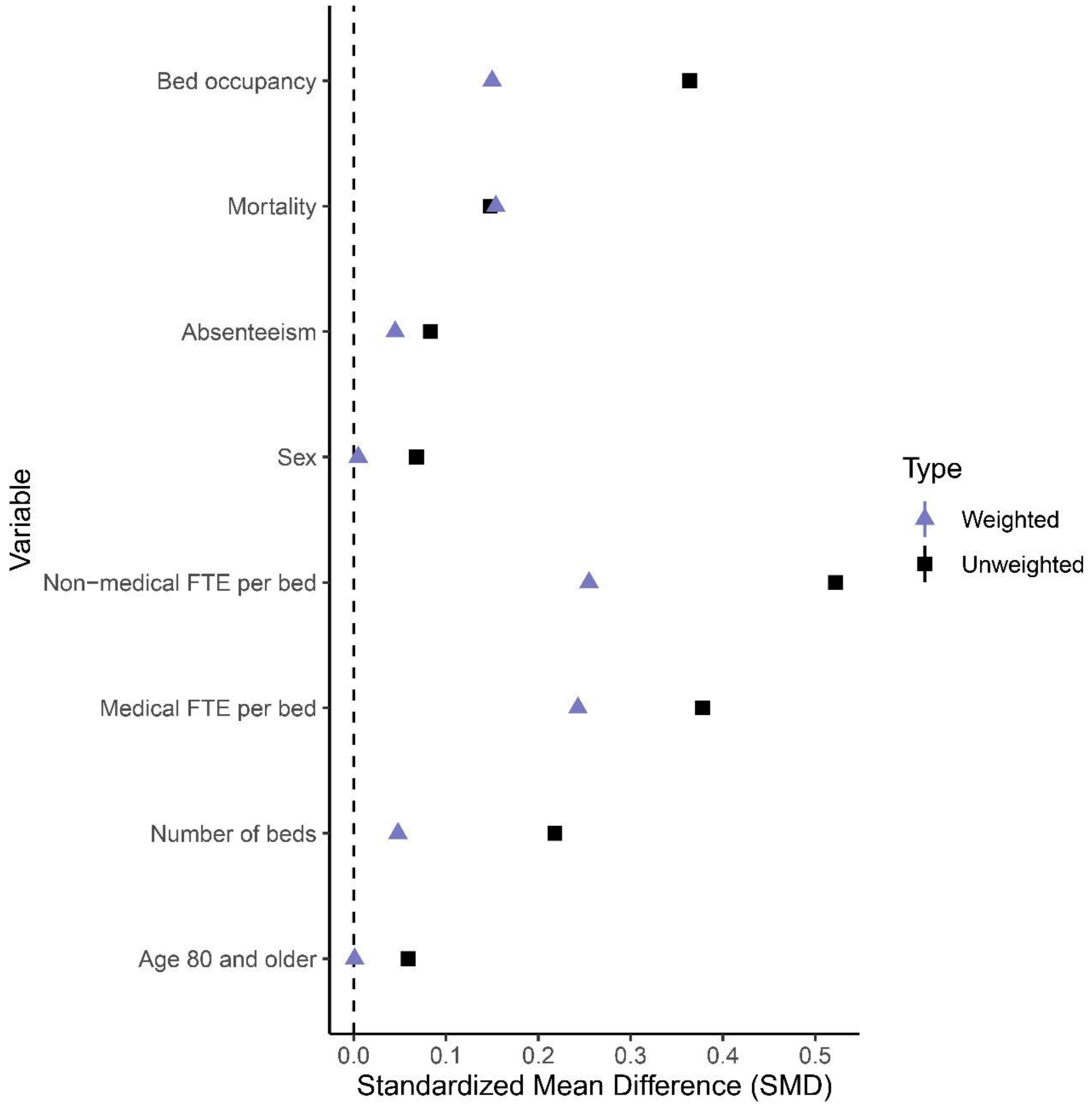

Table S3: Supplementary anticholinergic prescription indicators (aggregated over 18-month periods).

	Intervention		Control	
**Group**	**Before**	**After**	**Before**	**After**
**Number of distinct patients[a]**	1656	1706	3499	3632
**Number of prescriptions[a]**	139 578	130 657	320 284	319 289
**Distinct patients with anticholinergic medication[a]**	210	154	500	467
**Total anticholinergic prescriptions[a]**	1871	1239	5152	4714
**Proportion of patients with ≥1 anticholinergic[a]**	0.127	0.090	0.143	0.129
**Average number of anticholinergics per resident[a]**	1.13	0.73	1.47	1.30
**Average number of prescriptions per resident per month[a]**	4.68	4.25	5.09	4.88

a Aggregated over 18 months before and after intervention.

Availability of Study Material

The booklet describing the appropriate use of medications among older nursing home residents (Livret du medicament: Pour un bon usage chez la personne âgée; 3° edition; 2016) is available in French on request from the corresponding author or from the Korian Foundation.

Conversely, the aid for substitution of drugs, and the list of alternative drugs is a proprietary tool developed by the Korian group. Data can be made available on reasonable written request to the corresponding author.

Table S4 : STROBE Statement—checklist of items that should be included in reports of observational studies

	Item No.	Recommendation	Page No.
**Title and abstract**	1	(*a*) Indicate the study's design with a commonly used term in the title or the abstract	1 (abstract)
		(*b*) Provide in the abstract an informative and balanced summary of what was done and what was found	1 (abstract)
**Introduction**			
Background/rationale	2	Explain the scientific background and rationale for the investigation being reported	1-2
Objectives	3	State specific objectives, including any prespecified hypotheses	2
**Methods**			
Study design	4	Present key elements of study design early in the paper	2
Setting	5	Describe the setting, locations, and relevant dates, including periods of recruitment, exposure, follow-up, and data collection	2-4
Participants	6	(*a*) *Cohort study*—Give the eligibility criteria, and the sources and methods of selection of participants. Describe methods of follow-up	2-4
Variables	7	Clearly define all outcomes, exposures, predictors, potential confounders, and effect modifiers. Give diagnostic criteria, if applicable	2-4
Data sources/ measurement	8*	For each variable of interest, give sources of data and details of methods of assessment (measurement). Describe comparability of assessment methods if there is more than one group	4-5
Bias	9	Describe any efforts to address potential sources of bias	5
Study size	10	Explain how the study size was arrived at	Not applicable

Continued on next page

Quantitative variables	11	Explain how quantitative variables were handled in the analyses. If applicable, describe which groupings were chosen and why	4-5
Statistical methods	12	(*a*) Describe all statistical methods, including those used to control for confounding	5
		(*b*) Describe any methods used to examine subgroups and interactions	N/A
		(*c*) Explain how missing data were addressed	5
		(*d*) *Cohort study*—If applicable, explain how loss to follow-up was addressed	N/A
		(*e*) Describe any sensitivity analyses	N/A
Participants	13*	(a) Report numbers of individuals at each stage of study—eg numbers potentially eligible, examined for eligibility, confirmed eligible, included in the study, completing follow-up, and analysed	5-6
		(b) Give reasons for non-participation at each stage	6
		(c) Consider use of a flow diagram	Figure 1, p6
Descriptive data	14*	(a) Give characteristics of study participants (eg demographic, clinical, social) and information on exposures and potential confounders	Table 1, p6-7
		(b) Indicate number of participants with missing data for each variable of interest	6
		(c) *Cohort study*—Summarise follow-up time (eg, average and total amount)	5-7, Figure 2
Outcome data	15*	*Cohort study*—Report numbers of outcome events or summary measures over time	5-7, Figure 2
Main results	16	(*a*) Give unadjusted estimates and, if applicable, confounder-adjusted estimates and their precision (eg, 95% confidence interval). Make clear which confounders were adjusted for and why they were included	7-8
		(*b*) Report category boundaries when continuous variables were categorized	7-8
		(*c*) If relevant, consider translating estimates of relative risk into absolute risk for a meaningful time period	7-8

Continued on next page

Other analyses	17	Report other analyses done—eg analyses of subgroups and interactions, and sensitivity analyses	N/A
Key results	18	Summarise key results with reference to study objectives	5-8
Limitations	19	Discuss limitations of the study, taking into account sources of potential bias or imprecision. Discuss both direction and magnitude of any potential bias	5-6
Interpretation	20	Give a cautious overall interpretation of results considering objectives, limitations, multiplicity of analyses, results from similar studies, and other relevant evidence	10
Generalisability	21	Discuss the generalisability (external validity) of the study results	10
**Other information**			
Funding	22	Give the source of funding and the role of the funders for the present study and, if applicable, for the original study on which the present article is based	10
